# An Analytical Approach for Dispatch Operations of Emergency Medical Services: A Case Study of COVID-19

**DOI:** 10.1007/s43069-023-00218-3

**Published:** 2023-05-19

**Authors:** Jing Liu, Ruilin Ouyang, Chun-An Chou, Jacqueline Griffin

**Affiliations:** Mechanical and Industrial Engineering, Northeastern University, Boston, MA 02115 USA

**Keywords:** Emergency medical services, Resource allocation, Ambulance dispatch, COVID-19, Optimization modeling, Simulation Analysis

## Abstract

**Abstract:**

Emergency medical services (EMS) aims to deliver timely ambulatory care to incidents in communities. However, the operations of EMS may contend with suddenly increasing demands resulting from unexpected disasters such as disease outbreaks (e.g., COVID-19) or hurricanes. To this end, it usually requires better strategical decisions to dispatch, allocate, and reallocate EMS resources to meet the demand changes over time in terms of demographic and geographic distribution of incidents. In this study, we focus on the operation of the EMS resources (i.e., ambulance dispatch) in response to a demand disruption amid the COVID-19 pandemic. Specifically, we present a analytical framework to (1) analyze the underlying demographic and geographic patterns of emergency incidents and EMS resources; (2) develop a mathematical programming model to identify potential demand gaps of EMS coverage across different districts; and (3) provide a remedial reallocation solution to the EMS system with the existing ambulance capacity. The proposed method is validated with emergency response incident data in New York City for the first COVID-19 surge from March to April 2020. We found that it takes a long incident response time to scenes which reflects unexpected incident demands during COVID-19 surge. To cover such disruptive demands, ambulances need to be reallocated between service districts while meeting the response time standard. The proposed framework can be potentially applied to similar disruptive scenarios in the future and other operational systems disrupted by other disasters.

**Highlights:**

- We propose an analytical framework using optimization modeling and simulation techniques for EMS resource allocation in response to a demand disruption amid the COVID-19 pandemic.
- We propose mathematical programming models to identify potential demand gaps of EMS coverage across different districts.
- We provide a remedial reallocation solution to the EMS system with the existing ambulance capacity.

## Introduction

As the first responder to critically ill patients, emergency medical services (EMS) aims to deliver timely critical prehospital care with necessary treatment and support to life-threatening incidents in communities [1]. The operations of EMS may contend with suddenly increasing demands resulting from unexpected disasters such as disease outbreaks (e.g., COVID-19) or hurricanes. Accordingly, the EMS response time for emergency medical personnel to arrive at the scene of “life-threatening” cases has significantly increased in recent years [2, 3]. In particular, large populations could make EMS systems more vulnerable to such unexpected disasters.

As the most populous and densely populated city in the United States, New York City (NYC) needs to provide an EMS system to serve more than 8.2 million people. In the early stage of the COVID-19 outbreak in March 2020, the number of diagnosed coronavirus cases increased exponentially with the accompanying surge in ambulance demand. The NYC’s EMS received 30,000 more calls in one month, from March 16 to April 15, 2020, compared to the same period last year. On March 30, 2020, there was a 60% increase in calls compared to the same date last year. The unprecedented number of emergency calls led to a surge in EMS response time, which result in insufficient ambulances to serve these demands, and therefore patients had to wait. According to the reported statistics [4], the average daily ambulance response time during the peak demand period (March 16 to April 15) of COVID-19 in NYC increased from 10.4 min to 17.8 min, 7 min slower than in 2019. The average response time for high-risk calls increased by 3 min, while for low-risk calls it increased by 11 min. NYC’s EMS, facing with high demand for emergency calls, arrived too late for ambulances to reach patients, which might not save some patients with life-threatening conditions, especially cardiac arrest and choking patients [5, 6]. In previous studies of cardiac arrest, ambulance response time even had a significant impact on patient hospital discharge time [7]. Patients with a response time of more than 8 min are twice as likely to eventually die as patients with a response time of less than 8 min [8]. The difference between a minute or two of response time may be the difference between survival and death. The success rate and survival rate of resuscitation patients are inversely proportional to the length of the ambulance response time [7].

In this study, our goal is to analyze the NYC’s EMS operating system (i.e., EMS dispatch time and location) in response to huge incident demands COVID-19 pandemic. Among four major impacts on the response time including (1) people issues; (2) operational practices (e.g., dynamic deployment planning and matching of supply and demand); (3) performance information; and (4) supporting materials [9], we mainly focus on operational practices, including ambulance allocation-reallocation and ambulance storage that can match supply and demand. Specifically, we formulate the EMS resources allocation and reallocation as optimization problems and provide an analytical framework using mathematical programming and simulation analysis techniques, which aims to narrow the gap between huge demand and short supply within acceptable response time. Firstly, we develop a mathematical model to minimize the gap between EMS supply and incident demand through allocating sufficient and optimal EMS resources to the EMS stations in units of ambulance dispatch with the minimum travel distance. Furthermore, we implement scenario analysis, including priorities, consideration of the five boroughs as a whole versus taking each borough separately, and different measures of travel time. Subsequently, we develop an assignment model to determine the shortage of dispatches between boroughs and stations with the minimum travel distance to meet the optimal dispatch solution. Finally, we employ simulation analysis to identify the number of ambulances each station should be equipped to match the optimal dispatch allocation within acceptable response time.

Our contributions are to (1) comprehensively study NYC’s EMS operations during COVID-19 by analyzing emergency incidents (from NYC Open Data) and EMS resources; (2) propose an analytical framework for jointly considering EMS resource allocation and response time; and (3) come up with strategical allocation and reallocation implementation in the face of suddenly increasing incident demands based on the number of additional ambulances needed and response time priorities.

The organization of this paper is as follows. In Sect. 2, we give a brief literature review on EMS allocation analysis. In Sect. 3, we describe the NY EMS dataset, including data characteristics and data analysis. In Sect. 4, we present the analytical framework to analyze the EMS dispatch operations by allocation/reallocation optimization and dispatch simulation analysis. In Sect. 5, we discuss the scenario analysis of the allocation model and display all the results of our framework compared with real EMS dispatch. In Sect. 6, we conclude the work and mention possible future work.

## Literature Review of Related Work

Many studies on ambulances discussed the impact of priorities on EMS system operations and the role of priority in ambulance assignment and ambulance response time problems is of great importance [10–12]. Marla et al. [11] pointed out that compared with low-priority patients, high-priority patients are more likely to abandon their rescue requests while waiting for EMS. Singer and Donoso [13] indicated that to improve ambulance response time, service could be denied for less serious calls, fleet size could be increased or cycle time could be reduced, and average ambulance travel time could be reduced. Acuna et al. [14] displayed a strategy to focus on patients with travel times longer than 15 min, proposing a bi-objective model to minimize the maximum time for priorities with travel times longer than 15 min. Based on the above studies, the role of priority in ambulance assignment and ambulance response time problems is of great importance. In the paper, we discuss the impact of priority on EMS operations in our scenario analysis of the ambulance allocation model and how it can be applied in our framework.

Ahmadi et al. [15] conducted a study on relief dispatching in critical disasters with the same consideration of disaster severity and priority, and in which equity of distribution is taken into account. Djamel et al. [16] discussed vehicle dispatching in a disaster, but differs from an ambulance as ordinary rescue vehicles in a disaster usually need to consider the maximum capacity of the vehicle, while ambulances usually can only aid one patient at a time and require strict sanitary conditions and medical accessories. Ordinary rescue vehicles can travel to and from multiple locations at once, while ambulances need to return to the hospital for follow-up operations, such as sterilization, after completing a rescue. Khalili-Damghani et al. [17] discussed the difficulty of nighttime EMS operations, as the cost of nighttime rescue operations is higher than daytime. In the simulation analysis section, we used a 24-hour call rate to cover the difference between daytime and nighttime.

For the main objective function, previous studies aimed to solve in mathematical model of EMS operation. Acuna et al. [14] minimizes the transfer and waiting time, and minimizes the maximum time by priority class when patients travel more than 15 min. Boutilier et al. [18] uses a robust optimization method with minimizing travel time to optimize the location and route of the ambulance under different transportation patterns considering uncertainty. In our mathematical model, our objective function also focuses on minimizing the ambulance travel time while minimizing the total number of resources allocated, the total cost of reallocating resources, and the balance between the gaps on accumulated travel time over boroughs.

Using a simulation-based optimization approach, Marla et al. [11] suggested that increasing ambulance vehicle fleet size and redesigning base locations can increase patient willingness to wait, thus increasing the probability of a successful ambulance service and minimizing the waste of resources caused by callers abandoning. Bélanger et al. [19] use a recursive simulation optimization framework to jointly solve the ambulance localization and real-time scheduling problems for static information. Singer et al. [13] quantified key performance indicators (e.g., average number of busy ambulances and average service rate) in association with queuing theory and evaluated options for improving ambulance response time. Wang [20] introduced an M/M/m queuing framework in the multi-period mixed integer programming model to calculate the response time based on the demand arrival and service rates for each area and the number of ambulances allocated to the affected area, where the demand rate follows an independent and identical Poisson distribution and the service rate follows an exponential distribution. In our study, using simulation analysis based on queue theory, we jointly consider allocation-reallocation, station capacity issues along with response time. In our simulation analysis, we inferred the minimum number of ambulances should be allocated to each area to meet the ambulance response time within an acceptable threshold based on the hourly demand arrival rate and service rate for each area, where the demand rate follows an independent and identical Poisson distribution and the service rate follows a normal distribution.

When Boutilier and Chan [18] considered travel time uncertainty, they use the random forest approach to predict the travel time for each path of different transportation modes. We consider travel path between EMS station and incidents locations. For the travel time measurements, we used the median of the historical travel time, as well as the estimated driving duration provided by Google Maps. According to Cookson et al. [21], EMS research needs to be associated with a focus on seriously ill patients, with special attention to the urgency or the identification of the individual. In addition, the performance of the method should be considered important. Our model considers the importance of each ambulance call and focuses on seriously ill and at-risk patients, i.e., high-priority patients.

Doan and Shaw [22] and Khalili-Damghani et al. [17] use stochastic optimization techniques to allocate resources in response to simultaneous disasters. Jagtenberg et al. [23] performed offline dispatching modeling (assuming that all incidents are known in advance ) and online ambulance dispatching (deciding the optimal ambulance to send to an incoming incident) for a realistic EMS system in the Netherlands. They demonstrated that when the online ambulance model dispatched the nearest idle vehicle to each incident, the EMS system obtained a late arrival ratio close to 2.7 times higher than the optimal offline policy. The main disadvantage of the nearest distance dispatch policy is that it possibly causes no nearest idle ambulance dispatches to upcoming incidents, leading to sub-optimal response time for the entire dispatch system. In this paper, we focus on the dynamic allocation of ambulances for the coming day or week, by assuming that daily ambulance demand calls and demand locations for the coming day or week are known in advance, and that we dispatch nearest ambulances to incidents with the shortest travel time. Thus, the nearest dispatch policy, we used in this paper, is optimal for the entire system in terms of response time.

## EMS Data Description and Analysis

This study mainly focuses on the EMS dispatch operations in New York City in response to the COVID-19 pandemic. The analysis and modeling in this study are based on the EMS Incident Dispatch Data, including incident location, incident start-time, incident types, incident travel time, incident response time (referred to as “response time”), total incident time, ambulance departure areas (i.e., ambulance stations), and ambulance arrival areas (boroughs) from the New York Open Data [24]. In particular, we retrieve the EMS data from 2015 to 2020, for which the data before the COVID-19 pandemic suddenly spread out in March 2020 is used as a baseline.

**Fig. 1:**
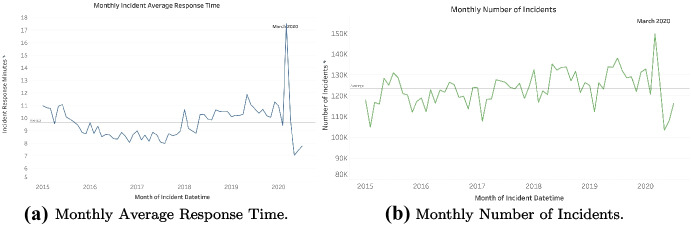
Trends of monthly average EMS response time and number of incidents from 2015 to 2020

According to the monthly incident volumes and response time, as shown in Fig. 1, COVID-19 pandemic caused a significant increase in EMS demands from March to April in 2020, which reached a high peak among the past six years, as well as ambulance response time (equivalently, patient wait time), overall medical service time, etc. For instance, the average response time between January and May from 2015 and 2019 is 8.24 min, whereas the average response time between March and April in 2020 is 9.82 min. Therefore, considering the given EMS capacity as usual, the ambulance supplies in NYC’s five boroughs do not sufficiently cover the incident demands in this sudden disaster surge. This motivates us to investigate the EMS operation preparedness in response to COVID-19.

In the following sections, we provide the details of geographic and demographic data analysis of EMS incidents and ambulance dispatch.

### Timeline of EMS Response to Incidents

**Fig. 2:**
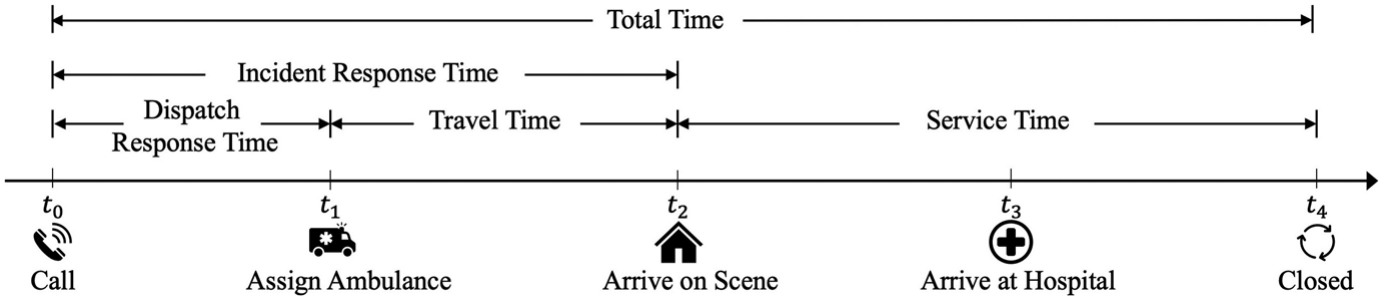
Timeline of EMS response to incidents

In Fig. 2, there are five time stamps defined as follows: $t_0$: the time of the incident is created; $t_1$: the time of the first ambulance is assigned; $t_2$: the time of an ambulance arrives at the location of the incident; $t_3$: the time of an ambulance arrives at the hospital; and $t_4$: the time of the incident is closed. Then, we define relevant time-interval variables as follows: dispatch response time $[ t_0$, $t_1]$, travel time $[t_1$, $t_2]$, incident response time $[t_0, t_2$], service time $[t_2, t_4]$, and total time $[t_0, t_4]$. It is observed that response time and dispatch response time are highly correlated with a coefficient = 0.8.

### EMS Incident Geographical Analysis

**Fig. 3:**
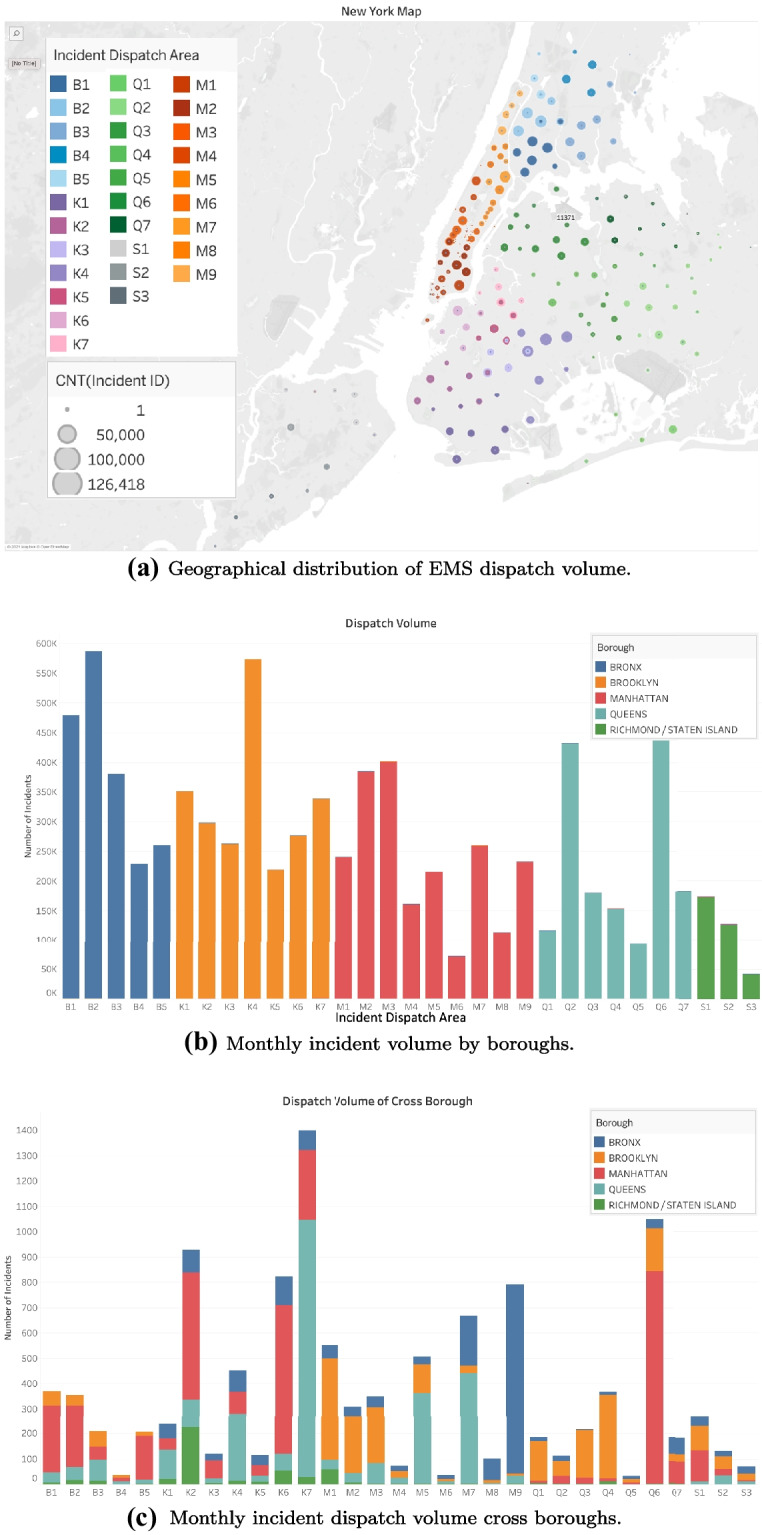
EMS incident dispatch distribution in New York City

The EMS incidents occur across five boroughs: {Brooklyn, Bronx, Manhattan, Queens, Staten Island} in New York, shown in Fig. 3a. There are in total EMS 39 stations where ambulances are located and dispatched: 7 in Brooklyn {$K_1,K_2,K_3,K_4,K_5,K_6,K_7$}; 5 in Bronx {$B_1,B_2,B_3,B_4,$ $B_5$}; 9 in the Manhattan {$M_1,M_2,M_3,M_4,M_5,M_6,M_7,M_8,M_9$}; 7 in Queens {$Q_1,Q_2,$ $Q_3,Q_4,$ $Q_5,Q_6,Q_7$}; 3 in Staten Island {$S_1,S_2,S_3$}; and 8 backup stations {$CW,T_1,T_2,$ $X_1,X_2,X_3,X_4,X_5$} that do not belong to any borough. Since the exact location of EMS stations is inferred from zip codes covered by the service. We assume that the destination units of EMS services are the boroughs (Bronx, Brooklyn, Manhattan, Queens, and Staten Island) and the departure units are the EMS stations (referred as “stations”).

For the travel time calculation, in this paper, we employ the following travel time measures to determine the trip distance: (1) median real travel time based on the last 5 months (January-May 2020); (2) median real travel time based on the past year (January-May 2019); and (3) real driving distance estimated based on zip codes using Google Maps.

Based on our analysis, we consider ambulance dispatch to be classified into three categories: same-area dispatch, cross-area dispatch, and backup dispatch. Among them, same-area dispatch is the primary dispatch method in EMS operations as shown in Fig. 3b. Specifically, we focus on the incidents pertaining same-area dispatch and cross-area dispatch in this study. For stations, we exclude the 8 backup stations in our study, since $\{CW,T_1,T_2,X_1,X_2,X_3,X_4,X_5\}$ have a very limited number of dispatch records throughout the year. Thus, in our further discussion, there are 31 stations in total. Figure 3a shows the incident area according to zip codes in New York City and the stations with coverage for ambulances dispatched in March and April 2020. Note that a zip code may be covered by more than one station while a single station may cover several zip codes. We then assume that the station covers the nearest group of zip codes, i.e., the shortest distance. This weighted group of covered zip codes is considered to be the approximate zip code of a station; thus allowing to obtain the “relative distance” between stations and boroughs. However, it does not indicate the exact distance between the station and the district, and is mainly used to compare the travel time between different stations and districts. Thus, we can assign available ambulances with the shortest travel time. There are two facts that support how to determine the availability of trips from stations to boroughs. The first is the number of free and activable ambulances at stations. The second is based on the most recent 2020 dispatch records that should have the same trip records to ensure trips are now available. Besides, we only consider dispatch records with response time less than its 97.5th percentile and total times less than its 97.5th percentile. We also consider calls canceled with their response time greater than its 2.5th percentile or total time greater than its 2.5th percentile. The data are summarized in Table 1.

**Table 1:** The summary of incident response time and total time (in minutes)

	Year (March and April)	2.5th	97.5th	Mean (Initial)	Standard Deviation (Initial)	Mean (Less than 97.5th Percentile)	Standard Deviation (Less than 97.5th Percentile)
Response Time	2015–2019	2.13	29.13	9.18	8.23	8.07	4.42
	2020	2.40	66.24	**13.98**	26.79	**9.82**	6.82
Total Time	2015–2019	10.93	120.35	65.52	28.72	63.12	25.84
	2020	12.08	148.00	68.96	39.13	64.08	27.69

### Type of EMS Incidents

**Fig. 4:**
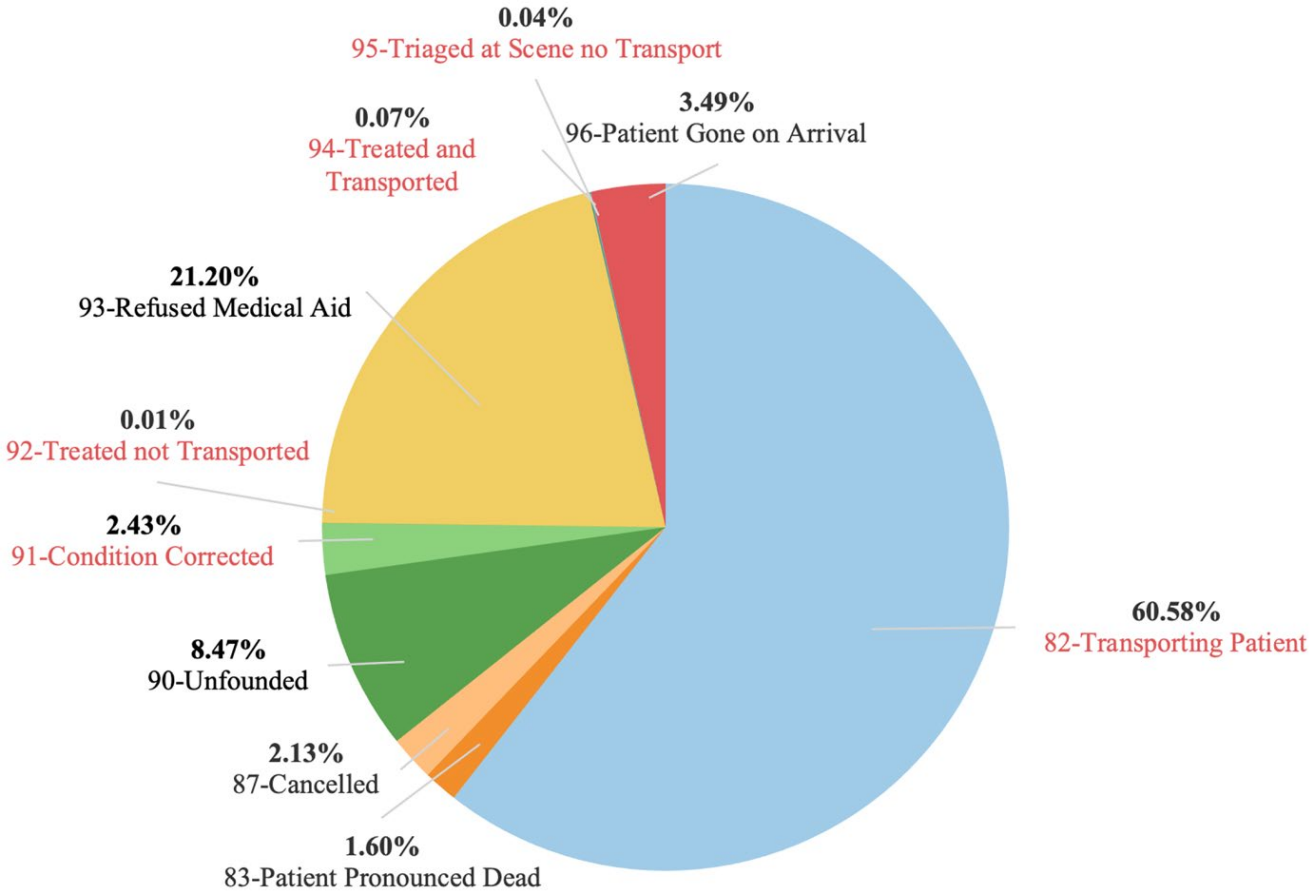
Incident type distribution in March and April 2020

There are 10 incident dispositions indicating the final outcome of incidents, as showed in Fig. 4, during March and April 2020. Among them, the two cases of “82: Transporting Patient” and “93: Refused Medical Aid” take 60.58% and 21.20% of the entire population, respectively. Besides, we refer other five incident dispositions “83: patient pronounced dead”, “87: cancelled”, “90: unfounded”, “93: refused medical aid”, and “96: patient gone on arrival” to “failure service calls” or “failure calls”. The remaining five incident dispositions “82: transporting patient”, “91: condition corrected”, “92: treated not transported”, “94: treated and transported”, and “95: triaged at scene no transport” are referred to as “successful service calls” or “successful calls”. In addition, we define cancel service calls with response time less than 2.5 percentile, total time less than 2.5 percentile, dispatch response time of 0, and incident disposition of “87: cancelled”. Figure 4 shows successful calls in red and failure calls in black.

We identify the emergency type using incident severity level code classification, where level 1 is the most severe and level 7 is least severe according to Table 2 [25]. We classify level 1 as “high-priority call” and others as “low-priority call”. As shown in Table 3, no significant difference in cancel call rates for priority, but a higher rate of failure calls for high priority than for low priority.

**Table 2:** Mean and median values of response time by incident severity level codes 2015–2020

Severity Level	1	2	3	4	5	6	7
Mean Value	5.11	6.85	6.77	8.56	8.92	9.21	11.08
Median Value	4.78	6.37	6.27	7.47	7.77	8.07	9.95

**Table 3:** Frequency of calls and failure rate by priority in March and April 2020

Emergency Type	Cancel Call Frequency	Cancel Rate	Failure Call Frequency	Failure Rate
High Priority	0.033	0.068	0.051	0.778
Low Priority	0.967	0.058	0.949	0.417

### EMS Incident Service Duration

**Fig. 5:**
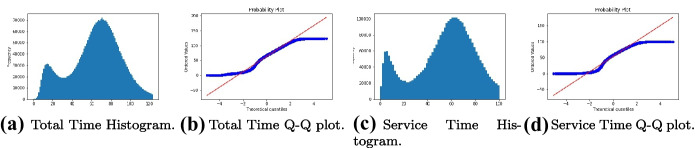
Histograms of total time and service time (in minutes) from 2015 to 2020

We define the total time as the time from the time of the incident was created to the end of the accident in Sect. 3.1. Figure 5a and b show the total time of EMS service duration is nearly normally distributed with mean values the standard deviation shown in Table 4. Similarly, we define the service time as the time from the ambulance arrival at the accident scene to the end of the accident in Sect. 3.1. Figure 5c and d show that the EMS service time is near normally distributed, for which mean service time and standard deviation are provided in Table 4.

**Table 4:** The average total time and service time (in minutes) in March and April in 2015–2020

	Mean Value	Standard Deviation		Mean Value	Standard Deviation
Service Time (2015–2019)	55.05	25.56	Service Time (2020)	54.26	26.93
Total Time (2015–2019)	63.12	25.84	Total Time (2020)	64.08	27.69

### Ambulance Travel Time Measurement and Trip Availability

In this study, we propose three ways to compute the ambulance travel time, which is defined as a time interval between the times of an ambulance first assigned and on scene:

1. Firstly, we consider the median travel time between the first ambulance assignment time and the first on scene based on incidents from January to May 2020. In Table 5, we present those median travel times between subregions in five different boroughs. We observed that travel times were greatly reduced due to the city lockdown from late March 2020 and travel times for some EMS dispatches were not recorded in March and April. Therefore we consider the median travel time from January to May 2020 to be the estimated travel time. In addition, we consider the high priority travel time and low priority travel time recorded in 2020 shown in Tables A17 and A18, respectively, in the Appendix.
2. Secondly, we consider the median travel time between the first assignment time and the first on scene in 2019, as shown in Table A19 in the Appendix because the ambulance travel time is in an ordinary circumstance without COVID 19.
3. Lastly, based on our assumption mentioned in Sect. 3.2, a station will cover the zip code group within the shortest distance, and the weighted combination of this group of zip codes is considered as the estimated zip code of the station. We then use zip code information to calculate driving distance and driving duration between boroughs and stations, as shown in Tables 6 and A16. The driving distance and driving duration are “relative distance” and is not equal to the exact distance between stations and boroughs. They are mainly used to compare the travel times between different stations and boroughs.

**Table 5:** Ambulance travel time (in minutes) in 2020

	B1	B2	B3	B4	B5	Q1	Q2	Q3	Q4	Q5	Q6	Q7	S1	S2	S3	
Bronx	**5**.**6**	**5**.**6**	**6**.**2**	**6**.**2**	**6**.**2**	0.0	13.1	0.0	3.4	4.2	6.7	8.0	10.7	0.0	17.8	
Brooklyn	8.9	9.4	9.6	0.0	0.0	**0**.**0**	**28**.**6**	**2**.**0**	**4**.**3**	**12**.**1**	**5**.**7**	**13**.**4**	4.9	3.3	14.9	
Manhattan	2.3	18.3	13.7	0.0	7.1	0.0	15.6	18.8	11.6	4.0	12.4	9.2	3.2	5.2	0.0	
Queens	10.8	7.3	4.5	0.0	0.0	5.2	5.4	6.2	6.2	6.1	6.2	5.6	0.0	19.8	0.0	
Staten Isalnd	0.0	20.6	0.0	0.0	0.0	0.0	0.0	0.0	0.0	0.0	0.0	0.0	**6**.**2**	**6**.**7**	**5**.**1**	

	K1	K2	K3	K4	K5	K6	K7	M1	M2	M3	M4	M5	M6	M7	M8	M9
Bronx	**15**.**1**	**11**.**8**	**4**.**6**	**7**.**6**	**10**.**9**	**8**.**5**	**0**.**0**	9.7	2.4	3.9	8.4	0.3	15.3	3.6	12.7	6.3
Brooklyn	6.4	5.1	5.7	6.3	7.3	4.9	6.4	7.9	3.3	9.0	6.7	7.8	0.0	9.9	10.1	0.0
Manhattan	10.1	7.7	5.5	5.6	12.2	3.8	8.1	**5**.**8**	**5**.**5**	**5**.**1**	**6**.**3**	**5**.**9**	**8**.**1**	**6**.**4**	**6**.**6**	**5**.**9**
Queens	7.1	4.6	8.9	2.2	6.8	10.6	3.7	0.0	4.1	5.3	3.8	11.9	0.0	6.6	0.0	9.9
Staten Island	11.5	3.4	19.5	0.0	0.0	6.6	28.5	1.6	0.0	4.7	0.0	7.4	0.0	0.0	0.0	0.0

**Table 6:** Driving duration (in minutes)

	B1	B2	B3	B4	B5	Q1	Q2	Q3	Q4	Q5	Q6	Q7	S1	S2	S3	
Bronx	**14**	**13**	**13**	**16**	**18**	45	31	25	25	24	21	23	45	45	50	
Brooklyn	44	40	42	45	45	45	41	38	31	30	27	40	35	38	43	
Manhattan	22	18	24	28	24	56	43	37	32	34	28	34	42	41	46	
Queens	30	29	22	25	32	**31**	**21**	**10**	**17**	**11**	**20**	**22**	43	46	51	
Staten Island	46	42	48	52	47	42	48	42	44	48	40	53	**12**	**1**	**13**	

	K1	K2	K3	K4	K5	K6	K7	M1	M2	M3	M4	M5	M6	M7	M8	M9
Bronx	47	44	44	32	38	35	31	31	26	27	20	19	18	15	18	13
Brooklyn	**30**	**24**	**14**	**22**	**11**	**14**	**21**	24	36	37	37	37	46	35	38	40
Manhattan	48	42	48	44	43	37	36	**26**	**15**	**15**	**16**	**14**	**12**	**21**	**20**	**12**
Queens	33	42	30	17	26	32	28	32	36	32	30	29	36	24	27	29
Staten Island	29	27	41	40	43	34	40	33	39	35	44	45	43	45	44	38

Firstly, we use zip code of incidents to find the latitude and longitude (geographic coordinates). Secondly, we use those geographic coordinates to calculate the weighted mean value of each borough and each station, and align them with zip codes. In some cases, the weighted mean value seams incorrect due to some cross dispatches, we use mode value of zip codes instead. Thirdly, as Google Map provides a good approximation of the driving distance between two zip codes in New York City, we use Google Map to gain driving distance and driving duration between borough zip codes and station zip codes. Note that the zip code of station is not exact location of stations, and the EMS data contains only the zip code of the accidents, not the zip code of the stations. We use the zip code information covered by the stations to approximately estimate the zip codes and location of the stations. Later, we use these information to measure the distance between stations and boroughs. Although, this measurement of ambulance travel distance might have a bias from the actual ambulance travel time, it helps us accurately estimate the distance relationship between stations and boroughs.

We use a non-zero travel time record in 2020 as an available dispatch trip from each station to each borough shown in Table 7. We consider the maximum daily dispatch volume record in 2020 as the maximum dispatch volume that can be served by the station per day shown in Table 8.

**Table 7:** Trip availability in 2020

	B1	B2	B3	B4	B5	Q1	Q2	Q3	Q4	Q5	Q6	Q7	S1	S2	S3	
Bronx	1	1	1	1	1	0	1	0	1	1	1	1	1	0	1	
Brooklyn	1	1	1	0	0	0	1	1	1	1	1	1	1	1	1	
Manhattan	1	1	1	0	1	0	1	1	1	1	1	1	1	1	0	
Queens	1	1	1	0	0	1	1	1	1	1	1	1	0	1	0	
Staten Island	0	1	0	0	0	0	0	0	0	0	0	0	1	1	1	

	K1	K2	K3	K4	K5	K6	K7	M1	M2	M3	M4	M5	M6	M7	M8	M9
Bronx	1	1	1	1	1	1	0	1	1	1	1	1	1	1	1	1
Brooklyn	1	1	1	1	1	1	1	1	1	1	1	1	0	1	1	0
Manhattan	1	1	1	1	1	1	1	1	1	1	1	1	1	1	1	1
Queens	1	1	1	1	1	1	1	0	1	1	1	1	0	1	0	1
Staten Island	1	1	1	0	0	1	1	1	0	1	0	1	0	0	0	0

**Table 8:** Maximum daily dispatch in 2020

Station	B1	B2	B3	B4	B5	Q1	Q2	Q3	Q4	Q5	Q6	Q7	S1	S2	S3	
Max Capacity	263	337	223	148	178	84	288	128	118	74	361	126	123	89	38	

Station	K1	K2	K3	K4	K5	K6	K7	M1	M2	M3	M4	M5	M6	M7	M8	M9
Max Capacity	232	218	205	336	148	180	210	154	242	294	101	127	52	156	77	192

## Analytical Framework for EMS Dispatch Operations

**Fig. 6:**
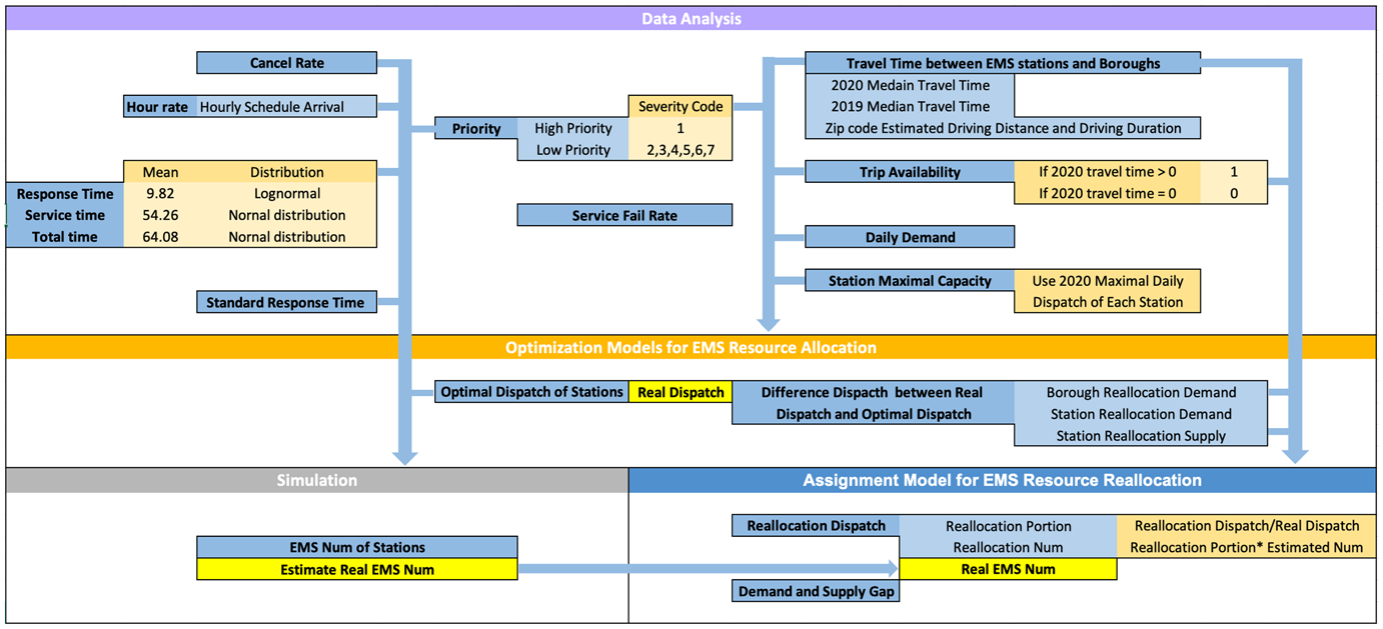
The diagram of our proposed analytical framework

In this section, we present an analytical approach to model and analyze the EMS incident dispatch operations such as resource allocation and reallocation problems. Specifically, we aim to provide an optimal EMS resource (ambulance) redistribution strategy to alleviate long ambulance response time in the disruptive EMS dispatch operations during the surge of COVID-19. The proposed framework is shown in Fig. 6. First, we develop mixed-integer linear optimization models to determine the EMS resource allocation (e.g., how many ambulances to be dispatched per time unit at individual EMS stations) in order to meet incident demands within their service areas. We assume the time unit is 24 h per day and 7 days per week. We count the total number of ambulance dispatches or runs in a day. It is noted that the ambulance response time is limited by the standard requirement (e.g., 5 min for high-priority case). Subsequently, we conduct extensive data analysis to estimate the real EMS arrival and service patterns (i.e., hourly arrival rate and average service time) as well as simulation analysis to capture the underlying ambulance volume based on the standard response time for individual EMS stations. By esimating the number of ambulances within an acceptable response time of 7 min at each station, we further estimate the daily average runs per ambulance, as well as the “real” number of ambulances at each station based on the real dispatch volume. Finally, we develop a mixed-integer linear optimization model to strategize EMS reallocation, that is, how to move around the dispatch volume and the number of ambulances across stations to meet the unexpected incident demand for different boroughs.

### Optimization Models for EMS Resource Allocation

In this section, we formally formulate the EMS resource allocation problem as an mixed-integer optimization model. The objective is to determine the optimal ambulance dispatch volume during the time period *T* of COVID-19 onset. Suppose there are |*I*| boroughs and $|K_i|$ stations in borough *i*. The notations of the sets, parameters, and variables are shown in Table 9.

**Table 9:** Notations of sets, parameters, and decision variables for EMS$\_$RA model

**Sets**	**Notation**
*I*	Set of boroughs.
*T*	Set of timestamps.
*K*	Set of stations.

**Parameters**	
$a_{k}$	Maximal resource (ambulances) at station *k* is available to serve (refer to Table 8).
$b_{k,i}$	Binary indicator to indicate if trip is established from station *k* to borough *i* (refer to Table 7).
$c_{i,t}$	Incident demand of borough *i* at time *t*.
$d_{k,i}$	Travel time from station *k* to borough *i*.
$\beta$	Cost of reallocating resources.
$\gamma$	Cost for unmet demands.
$\lambda$	Cost of ravel time.
$\mu$	Ratio to the maximal number of ambulance dispatched.

**Decision variables**	
$x_{k,t}$	The number of ambulance dispatched from station *k* at time *t*.
$w_{k,i,t}$	The number of ambulance dispatched to serve borough *i* from station *k* at time *t*.
$u_{i,t}$	Slack variable (unmet demands) to restrict the total number of resources being served in borough *i*
	must equal to the demand of borough *i* at time *t*.
$s_{k,t}$	Dummy variable greater than $|x_{k,\,t}-x_{k,\,{t\mathop{+}1}}|$.

The EMS resource allocation (EMS$\_$RA) model is formulated as follows:

1 $$\begin{aligned} \mathrm{(EMS\_RA)} \quad Min{}\quad & {} \beta \sum _{k\, \in\, K,\, t\, \in\, T/|T|} {|x_{k,\,t}-x_{k,\,{t\mathop{+}1}}|} + \lambda \sum _{k\, \in\, K, \ i\, \in\, I, \ t\, \in\, T}{d_{k,i} w_{k,\,i,\,t}} + \gamma \sum _{i\, \in\, I,\, t\,\in\, T}{u_{i,\,t}} \end{aligned}$$

2 $$\begin{aligned} s.t.{}\ & {} x_{k,\,t} = \sum _{i\, \in\, I}{w_{k,\,i,\,t}} \quad \forall \ k \in K, t \in T \end{aligned}$$

3 $$\begin{aligned}{} & {} \sum _{i\, \in\, I}{w_{k,\,i,\,t}} \le \mu a_{k} \quad \forall k \in K, t \in T \end{aligned}$$

4 $$\begin{aligned}{} & {} w_{k,\,i,\,t} \le \mu b_{k,\,i}a_{k} \quad \forall i \in I, k \in K, t \in T \end{aligned}$$

5 $$\begin{aligned}{} & {} c_{i,\,t} - u_{i,\,t} = \sum _{k\, \in\, K}{w_{k,\,i,\,t}} \quad \forall \ i \in I, \ t \in T \end{aligned}$$

6 $$\begin{aligned}{} & {} w_{k,\,i,\,t}, \ x_{k,\,t}, \ u_{i,\,t} \in \mathbb Z_{\ge\,0} \end{aligned}$$

The objective function in Eq. (1) minimizes the total cost of reallocating resources, total travel time, and unmet EMS incident demand. Constraint set in Eq. (2) indicates the number of resources served from station *k* equals the sum of the number of ambulance dispatched to all boroughs from station *k* at time *t*. Constraint set in Eq. (3) restricts the maximum number of resources serving from station *k* based on the value of $a_k$. Constraint set in Eq. (4) indicates the travel availability from station *k* to borough *i* at time *t*. Constraint set in Eq. (5) indicates that the total number of resources being served to borough equals to the incident demand minus the unmet demand of borough *i* at time *t*. all decision variables $w_{k,\,i,\,t}, \ x_{k,\,t}, \ u_{i,\,t}$ are constrained to be non-negative integers in Eq. (6). need to explain those parameters $\beta$, $\lambda$, and $\gamma$ in the objective function and how to set up their values.

We then propose to reformulate the EMS_RA model by linearizing the objective function as follows:

7 $$\begin{aligned} \mathrm{(EMS\_RA\_Linear)} \quad Min{}\quad & {} \beta \sum _{k\, \in\, K,\, t\, \in\, T/|T|}{s_{k,\,t}} + \lambda \sum _{k \,\in\, K,\,i\, \in \,I, \,t \,\in\, T}{d_{k,\,i} w_{k,\,i,\,t}} + \gamma \sum _{i\, \in\, I,\, t\,\in\, T}{u_{i,\,t}}\end{aligned}$$

8 $$\begin{aligned} s.t.{}\ & {} x_{k,\,t} = \sum _{i\, \in\, I}{w_{k,\,i,\,t}} \quad \forall k \in K, t \in T \end{aligned}$$

9 $$\begin{aligned}{} & {} \sum _{i\, \in\, I}{w_{k,\,i,\,t}} \le \mu a_{k} \quad \forall k \in K, t \in T \end{aligned}$$

10 $$\begin{aligned}{} & {} w_{k,\,i,\,t} \le \mu b_{k,\,i}a_{k} \quad \forall i \in I, k \in K, t \in T \end{aligned}$$

11 $$\begin{aligned}{} & {} c_{i,\,t} - u_{i,\,t} = \sum _{k\, \in\, K}{w_{k,\,i,\,t}} \quad \forall i \in I, t \in T \end{aligned}$$

12 $$\begin{aligned}{} & {} x_{k,\,t}-x_{k,\,{t\mathop{+}1}} \le s_{k,\,t} \quad \forall k \in K, t \in T/|T|\end{aligned}$$

13 $$\begin{aligned}{} & {} -x_{k,\,t}+x_{k,\,{t\mathop{+}1}} \le s_{k,\,t} \quad \forall k \in K, t \in T/|T|\end{aligned}$$

14 $$\begin{aligned}{} & {} w_{k,\,i,\,t}, \ x_{k,\,t}, \ u_{i,\,t}, \ s_{k,\,t} \in \mathbb Z_{\ge\, 0} \end{aligned}$$

The first term $\beta \sum _{k\, \in\, K,\,t \,\in\, T/|T|}{|x_{k,\,t}-x_{k,\,{t\mathop{+}1}}|}$ in the objective function is replaced with $\beta \sum _{k\, \in\, K,\,t\, \in \,T/|T|}{s_{k,\,t}}$ and the constraint sets in Eqs. (12) and (13). The rest of the model remains unchanged.

In practice, it is not likely to reallocate the EMS resource on a daily basis in response to the irregular changes in EMS incident demand across areas since it requires significant amount of workforce. Thus, we develop another EMS resource allocation model that allows for EMS resource reallocation on a weekly basis (dispatch volume as a unit of a day and a week as an allocation cycle). The notations of necessary parameters and decision variables are listed in Table 10.

**Table 10:** Notations of parameters and decision variables for the EMS$\_$RA$\_$Weekly model

**Parameters**	**Notation**
$\alpha$	Weight for the total number of resources assigned define what is $x_{k}$.
$\beta$	Weight for auxiliary variables $s_{k,\,t}$, i.e., daily reallocating resources.
$g_{k}$	Dispatch amount of last Monday at station *k* or average dispatch amount of last week at station k)
$\theta$	Weight for auxiliary variables $q_{k}$, i.e., weekly reallocating resources.

**Decision Variables**	
$x_{k}$	The number of resources assigned from station *k* at time *t*.
$u_{i,t}$	The number of demand not being satisfied in borough *i* at time *t*.
$q_{k}$	Auxiliary variable to indicate $|x_{k} - g_{k}|$.
$s_{k,t}$	Auxiliary variable to indicate $|\sum _{i\, \in\, I}{(w_{k,\,i,\,t}-w_{k,\,i,\,0})}|$.

The overall objective is still to minimize the total cost of reallocating resources, total travel time, and unmet EMS incident demand. The optimization model is given as follows:

15 $$\begin{aligned} \mathrm{(EMS\_RA\_Weekly)} \quad Min{} \quad& {} \alpha \sum _{k\, \in\, K,\, t\, \in\, T}{x_{k}} + \beta \sum _{k\, \in\, K,\,t\, \in\, T}{s_{k,\,t}} + \theta \sum _{k\, \in\, K}{q_{k}}\nonumber \\{} & {} + \lambda \sum _{k\, \in\, K,\,i\, \in \,I,\, t\, \in\, T}{d_{k,\,i} w_{k,\,i,\,t}}\end{aligned}$$

16 $$\begin{aligned} s.t.{}\ & {} x_{k} \ge \sum _{i\, \in\, I}{w_{k,\,i,\,t}} \quad \forall k \in K, t \in T \end{aligned}$$

17 $$\begin{aligned}{} & {} \sum _{i \,\in\, I}{w_{k,\,i,\,t}} \le \mu a_{k} \quad \forall k \in K, t \in T \end{aligned}$$

18 $$\begin{aligned}{} & {} w_{k,\,i,\,t} \le \mu b_{k,\,i}a_{k} \quad \forall k \in K,i \in I, t \in T \end{aligned}$$

19 $$\begin{aligned}{} & {} c_{i,\,t} - u_{i,\,t} = \sum _{k\, \in\, K}{w_{k,\,i,\,t}} \quad \forall i \in I, t \in T \end{aligned}$$

20 $$\begin{aligned}{} & {} \sum _{i\, \in\, I}{(w_{k,\,i,\,t}-w_{k,\,i,\,{0}})} \le s_{k,\,t} \quad \forall k \in K, t \in T\end{aligned}$$

21 $$\begin{aligned}{} & {} \sum _{i \,\in\, I}{(-w_{k,\,i.t}+w_{k,\,i,\,{0}})} \le s_{k,\,t} \quad \forall k \in K, t \in T\end{aligned}$$

22 $$\begin{aligned}{} & {} x_{k}-g_{k} \le q_{k} \quad \forall k \in K \end{aligned}$$

23 $$\begin{aligned}{} & {} -x_{k}+g_{k} \le q_{k} \quad \forall k \in K \end{aligned}$$

24 $$\begin{aligned}{} & {} x_{k}, \ u_{i,\,t}, \ q_{k}, \ s_{k,\,t}\in \mathbb Z_{\ge\, 0} \end{aligned}$$

The EMS$\_$RA$\_$Weekly model is similar as the EMS$\_$RA$\_$Linear model except that we force the EMS resource allocation plan to be fixed on a weekly basis, and minimize the gap of the EMS resource allocation plan between the current time horizon and previous time horizon. Constraint set in Eq. (16) indicates that the daily number of resources needed for station *k* in a week at time *t* less than to weekly scheduled number of resources that server all the boroughs in set *I* at time *t*. The constraint set in Eq. (24) limits the range of decision variables.

### EMS Dispatch Simulation

In this section, we construct a discrete-event simulation model to determine the number of ambulances that are needed to meet the standard response time. The standard response time, as mentioned in Sect. 3, are 5 min for high-priority calls and 9 min for low-priority calls, while the standard response time is 7 min without considering priority. In simulation modeling, for each station, we need to estimate the call arrival process and EMS service time.

**Fig. 7:**
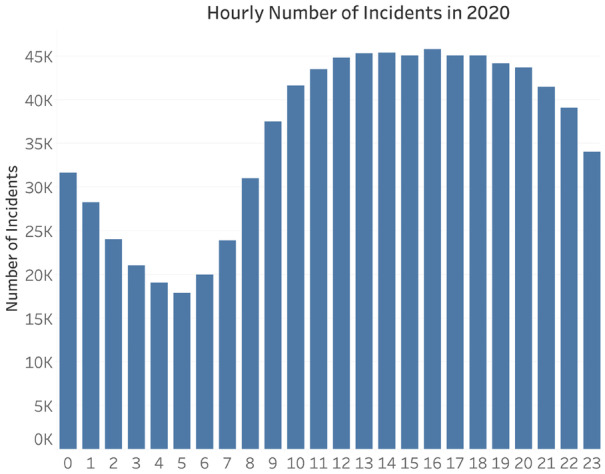
The trend of hourly incidents in 2020

The dispatch volume for each station is derived from the result of the EMS-RA model in Sect. 4.1. We define EMS service time as the time interval between the ambulance arrival at the accident scene and the end of the accident. According to our analysis, the EMS service time in March and April 2020 is near normally distributed, as shown in Fig. 5 in Sect. 3.4. The mean EMS service time is 54.26 min with a standard deviation of 26.93 min. Similarly, the EMS service time of each station is also normally distributed; mean EMS service times with standard deviation are shown in Table A21 in the Appendix. Then, we consider EMS calls as arrival events in an hourly basis as shown in Fig. 7. In Table 11, the hourly dispatch rate is inferred from the percentage of hourly dispatched volume out of the total dispatched volume.

The average incident response time value of regular period from 2015 to 2019 is 8.24 min. As the demand increases significantly in March and April in 2020, the average incident response time increased to 9.82 min, which exceeds the standard EMS response time of 7 min. The significant increased incident response time also indicates that the number of existing ambulances is insufficient for EMS incident demand.

Through simulation analysis, we can observe the variation in response time by the number of ambulances for the same call arrival rate and service time distribution. The intuitive idea is that as the number of ambulances increases, the average response time decreases. In simulation analysis, we then vary the number of ambulances at each station and constrain the response time less than the standard response time with acceptable utility rate (minimizing EMS resources). By setting the most acceptable number of ambulances at each station, we can approximate the daily average dispatches of each ambulance and the initial number of ambulances at each station.

**Table 11:** Hourly EMS resource dispatch rate in 2020. (%)

Hour	Daily Percentage	Hour	Daily Percentage	Hour	Daily Percentage	Hour	Daily Percentage
1	3.53	7	2.48	13	5.22	19	5.13
2	3.12	8	2.92	14	5.07	20	5.15
3	2.78	9	3.81	15	5.02	21	4.61
4	2.52	10	4.65	16	5.30	22	4.27
5	2.37	11	5.13	17	5.17	23	3.75
6	2.27	12	5.28	18	5.21	0	3.94

### Assignment Model for EMS Resource Reallocation

In any situation where there is gap between EMS dispatches and EMS incidents within service district, we present an assignment optimization model to reallocate EMS resources among service districts to fill the supply–demand gap. However, in the meantime, we would like to ensure a minimum EMS reallocation. The notations of parameters and decision variables are shown in Table 12.

**Table 12:** Notations of parameters and decision variables for EMS$\_$RR model

**Parameters**	**Notation**
$r_{i}$	Reallocation demand of borough *i* at time *t*.
$p_{k}$	Maximal resource at station *k* is able to reallocate.
$b_{ki}$	Trip availability from station *k* to borough *i*.
$v_{ki}$	Travel time serving borough *i* from dispatch *k*.
$\alpha$	Weight for number of resources.
$\lambda$	Weight for travel time assigned to different boroughs.
$\gamma$	Weight for the balance of travel time between different boroughs.

**Decision Variables**	
$x_{ki}$	number of resources reallocated from station *k* to borough *i*.
$y_{i}$	gap between reallocation demand and reallocation supply for borough *i*.

The mathematical formulation of the EMS resource reallocation (EMS$\_$RR) model is presented as follows:

25 $$\begin{aligned} \mathrm{(EMS\_RR)} \quad Min{} &\quad {} \alpha \sum _{k\, \in\, K,\, i\, \in\, i}{x_{ki}} + \gamma \sum _{i\, \in\, i}{y_{ki}} + \lambda \sum _{k\, \in\, K,\,i \,\in\, I}{v_{ki} x_{ki}} \end{aligned}$$

26 $$\begin{aligned} s.t.{} & {} \sum _{i\, \in\, I}{x_{ki}} \le p_{k} \quad \forall k \in K \end{aligned}$$

27 $$\begin{aligned}{} & {} \sum _{k\, \in\, K}{x_{ki}} = r_{i} - y_{i} \quad \forall i \in I \end{aligned}$$

28 $$\begin{aligned}{} & {} {x_{ki}} \le b_{ki}p_{k} \quad \forall i \in I \forall k \in K \end{aligned}$$

29 $$\begin{aligned}{} & {} x_{ki}, \ y_{i}\in \mathbb Z_{\ge\, 0} \end{aligned}$$

The objective function in Eq. (25) minimizes the total number of resources to reallocate, the total gap between EMS demand and supply for all boroughs, and the total travel time spent on reallocation. The constraint set in Eq. (26) restricts the maximum number of resources that can be reallocated from borough *i*. The constraint set in Eq. (27) calculates the gap between reallocation demand and reallocation supply for borough *i*. The constraint set in Eq. (28) indicates the availability of reallocating resources from station *k* to *i*. The constraint set in Eq. (29) limits the range of decision variables.

## Experimental Results

### EMS Allocation Model

In this section, we show the optimized dispatch allocation for 31 stations. For 61 days from March 1st to April 30th, the dispatch allocation result is shown in Fig. A12. We compare the average number of dispatch from EMS_RA_Linear model with the real dispatch number in the New York Open Data for those 61 days. The difference between our model and real dispatch is shown in Fig. A13. The average daily dispatch volume and difference volume between our model and real dispatch are shown in Table 8.

The average daily delivery demand is 3,979 for the two months from March to April 2021. As shown in Fig. A13, we obtain the optimal dispatch volume per day for each station from model $EMS\_RA$ and calculate the difference with the real 61-day average dispatch volume for each station. We compare the difference between our dispatch outcome of 3 different travel time measures. The mean absolute error for the dispatch volume of 3 different travel time measurement “median travel time in 2020”, “median travel time in 2019”, and “driving duration” are 63.03, 57.64, 57.00 respectively. The whiter cell means the difference is more close to zero, a more red cell means the more difference between allocation result and actual dispatch. It shows that the dispatch outcomes $x_k$ obtained using the driving duration as travel time measurement can be most close to the actual dispatch. It is best to use the driving duration as the estimated travel time measurement.

**Fig. 8:**
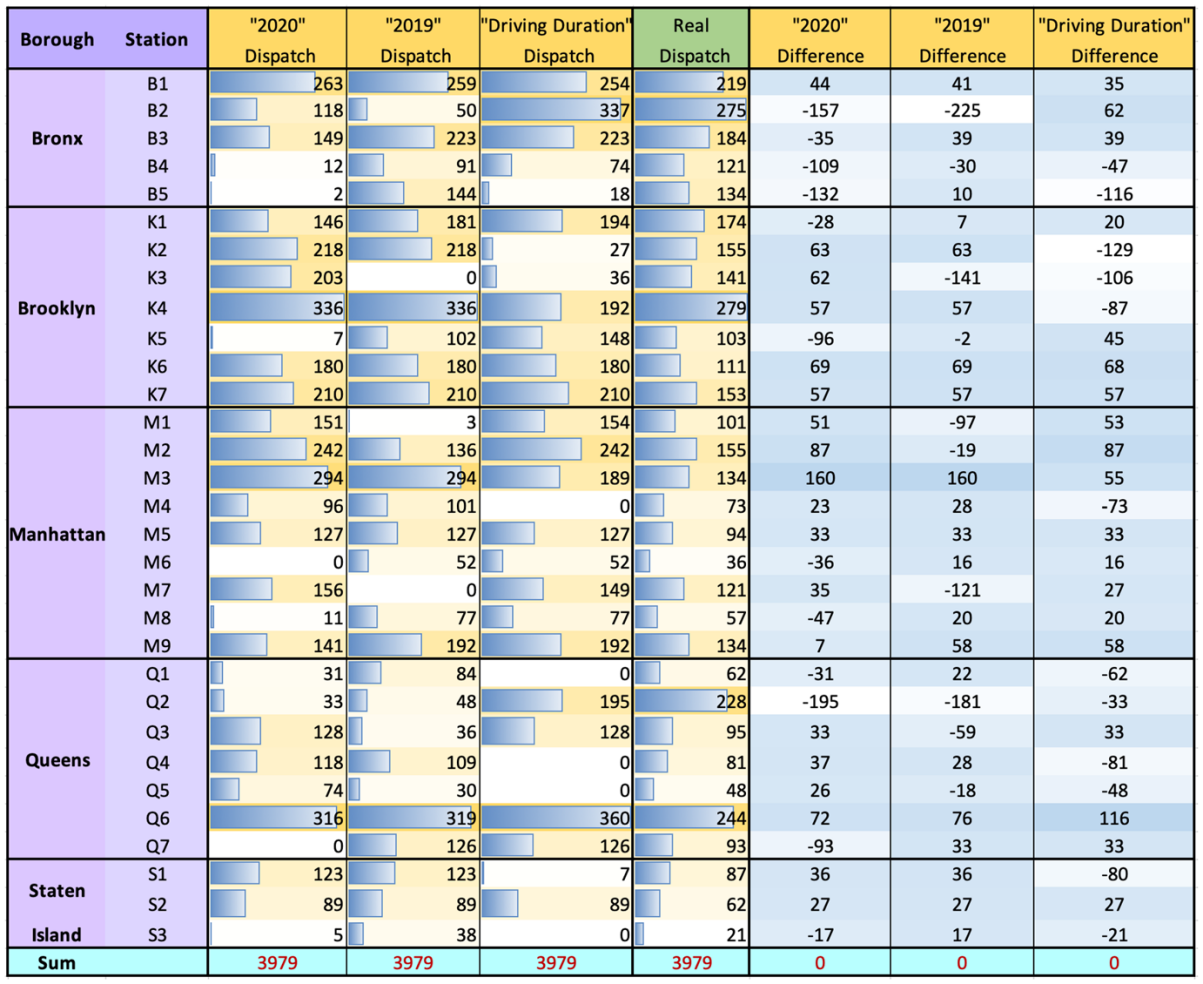
Statistics of daily EMS incident dispatch across five service districts in NYC

### Scenario Analysis for EMS Allocation Model

In this section, we consider the different scenarios that can be covered by our EMS allocation model $EMS\_RA$ in Sect. 4.1. We use driving duration as travel time measurement in this section. When comparing the daily and weekly EMS resource allocation plan, we can see a clear difference between the results of the daily and weekly models in Table 13. For priority, we obtained the results by considering high and low priority demands separately, see Table 14. For geographic location, we display different results considering each district separately, or all five districts at the same time in Table 14. The results we obtained show that our allocation model can be used in different scenarios depending on the research needs. After obtaining the results of the assignment model, it is thus possible to analyze and reassign the model by simulation to obtain results as in Sects. 5.3 and 5.4.

**Table 13:** Daily and weekly EMS dispatch volumes from the $EMS\_RA$ model and $EMS\_RA\_Weekly$ (one day or one week as an allocation cycle, dispatch volume as a unit of a day). The total volumes of daily dispatches on $EMS\_RA$ model and $EMS\_RA\_Weekly$ model are 4,401 and 3,979, respectively. Use driving duration as travel time measurement

	B1	B2	B3	B4	B5	Q1	Q2	Q3	Q4	Q5	Q6	Q7	S1	S2	S3	
Daily	254	337	223	74	18	0	195	128	0	0	360	126	7	89	0	
Weekly	263	337	223	143	19	0	286	128	0	0	361	126	0	89	0	

	K1	K2	K3	K4	K5	K6	K7	M1	M2	M3	M4	M5	M6	M7	M8	M9
Daily	194	27	36	192	148	180	210	154	242	189	0	127	52	149	77	192
Weekly	232	26	68	299	148	180	210	154	242	264	0	127	52	156	77	192

**Table 14:** High and low priority average daily EMS dispatch volumes from the $EMS\_RA$ model. The total volumes of daily dispatches is 3,980. Use driving duration as travel time measurement

Station	Low Priority	High Priority	Station	Low Priority	High Priority
B1	240	5	K1	189	9
B2	310	10	K2	44	6
B3	211	0	K3	66	2
B4	89	4	K4	210	16
B5	22	1	K5	140	0
Q1	0	0	K6	165	6
Q2	196	6	K7	188	11
Q3	121	0	M1	145	1
Q4	4	0	M2	229	0
Q5	1	0	M3	187	8
Q6	342	0	M4	7	0
Q7	108	11	M5	120	0
S1	13	1	M6	45	4
S2	82	2	M7	140	5
S3	2	0	M8	68	5
			M9	182	0

**Table 15:** Average daily EMS dispatch volumes from the $EMS\_RA$ model. The total volumes of daily dispatches is 3,039 for five boroughs respectively. Use “2020 travel time” travel time measurement

	Manhattan	Queens	Staten_Island	Bronx	Brooklyn	**Sum**	**All**		Manhattan	Queens	Staten_Island	Bronx	Brooklyn	**Sum**	**All**
B1	0	0	0	227	0	**227**	**254**	K1	0	0	0	0	251	**252**	**194**
B2	0	1	0	198	1	**201**	**337**	K2	0	2	0	0	17	**19**	**27**
B3	0	0	0	237	0	**237**	**223**	K3	0	0	0	0	204	**204**	**36**
B4	0	0	0	155	0	**155**	**74**	K4	0	1	0	0	124	**125**	**192**
B5	0	0	0	142	0	**142**	**18**	K5	0	0	0	0	158	**158**	**148**
Q1	0	36	0	0	0	**36**	**0**	K6	0	0	0	1	168	**169**	**180**
Q2	0	194	0	0	0	**194**	**195**	K7	0	2	1	0	182	**185**	**210**
Q3	0	11	0	0	3	**14**	**128**	M1	137	0	1	0	3	**141**	**154**
Q4	0	116	0	0	2	**118**	**0**	M2	252	1	0	0	2	**255**	**242**
Q5	0	64	0	0	0	**64**	**0**	M3	223	1	0	1	0	**225**	**189**
Q6	0	254	0	0	2	**256**	**360**	M4	0	0	0	0	0	**0**	**0**
Q7	0	124	0	0	0	**124**	**126**	M5	0	0	0	0	0	**0**	**127**
S1	0	0	80	0	1	**81**	**7**	M6	52	0	0	0	0	**52**	**52**
S2	0	0	45	0	0	**45**	**89**	M7	82	0	0	1	0	**83**	**149**
S3	0	0	35	0	0	**35**	**0**	M8	0	0	0	2	0	**2**	**77**
								M9	116	0	0	0	0	**116**	**192**

We demonstrate the results of the simulation analysis in Sect. 5.3 and the reallocation model in Sect. 5.4, using driving duration as the measure of travel time with one day as an allocation cycle, dispatch volume as a unit of a day without considering priority (see Table 15).

### Simulation Analysis

**Fig. 9:**
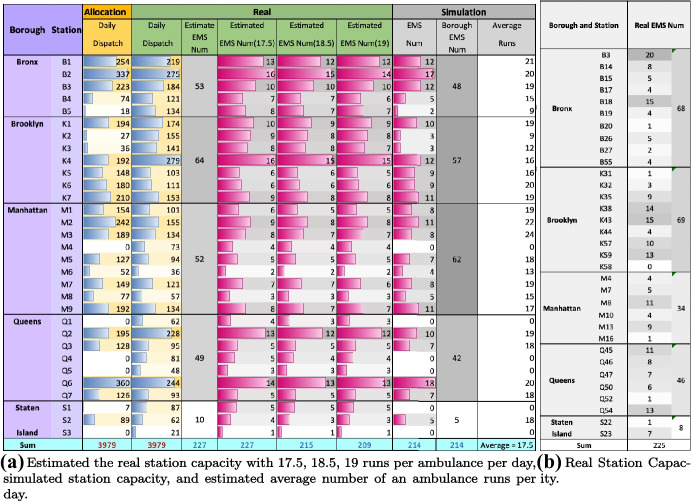
Simulation result and real station capacity

We attempt to infer the number of ambulances that each stations have to meet the standard response time using simulation analysis by given the number of station dispatch for each station in Sect. 5.1. We use average EMS response time 7 min as our standard response time. In Fig. 9, we display the number of ambulances capacity that each stations should have in order to meet dispatch demand and standard response time. As the simulation result, there are 214 ambulances in total. For each ambulance, we can infer that the average dispatch of each ambulance is 17.5 runs per day, which means an ambulance should serve 17.5 calls in average per day to meet a given dispatch demand with a response time of less than 7 min. Similarity, for real dispatch of ambulances, we attempt to infer the number of ambulances capacity of each station. The total number of ambulances is 227 by given 17.5 runs per ambulance per day; the total number of ambulances is 221 by given 18 runs per ambulance per day; the total number of ambulances is 215 by given 18.5 times per ambulance per day; the total number of ambulances is 209 as well as by given 19 times per ambulance per day assuming more tense the situation will be. The “Fire Department of the City of New York” [26] gives New York EMS station locations and the number of ambulance capacity each station has, which has difference records from our data records. Nevertheless, the total number of ambulances in New York city is 225 in Fig. 9. In short, NYC EMS stations need 214 ambulances in total to meet the dispatch demand with a response time of less than 7 min. The less an ambulance can serve, the shorter response time can be, and less usage rate. The more an ambulance can serve, the longer response time can be. Therefore, we need to make a trade-off between usage rate and response time.

### EMS Reallocation

In this section, we display the number of dispatches as well as ambulances at each station should be reallocated to meet the requirement in Sects. 4.1 and 4.3, given the existing resources (the total number of dispatches in the record and the total number of ambulances we inferred in Sect. 5.3.

**Fig. 10:**
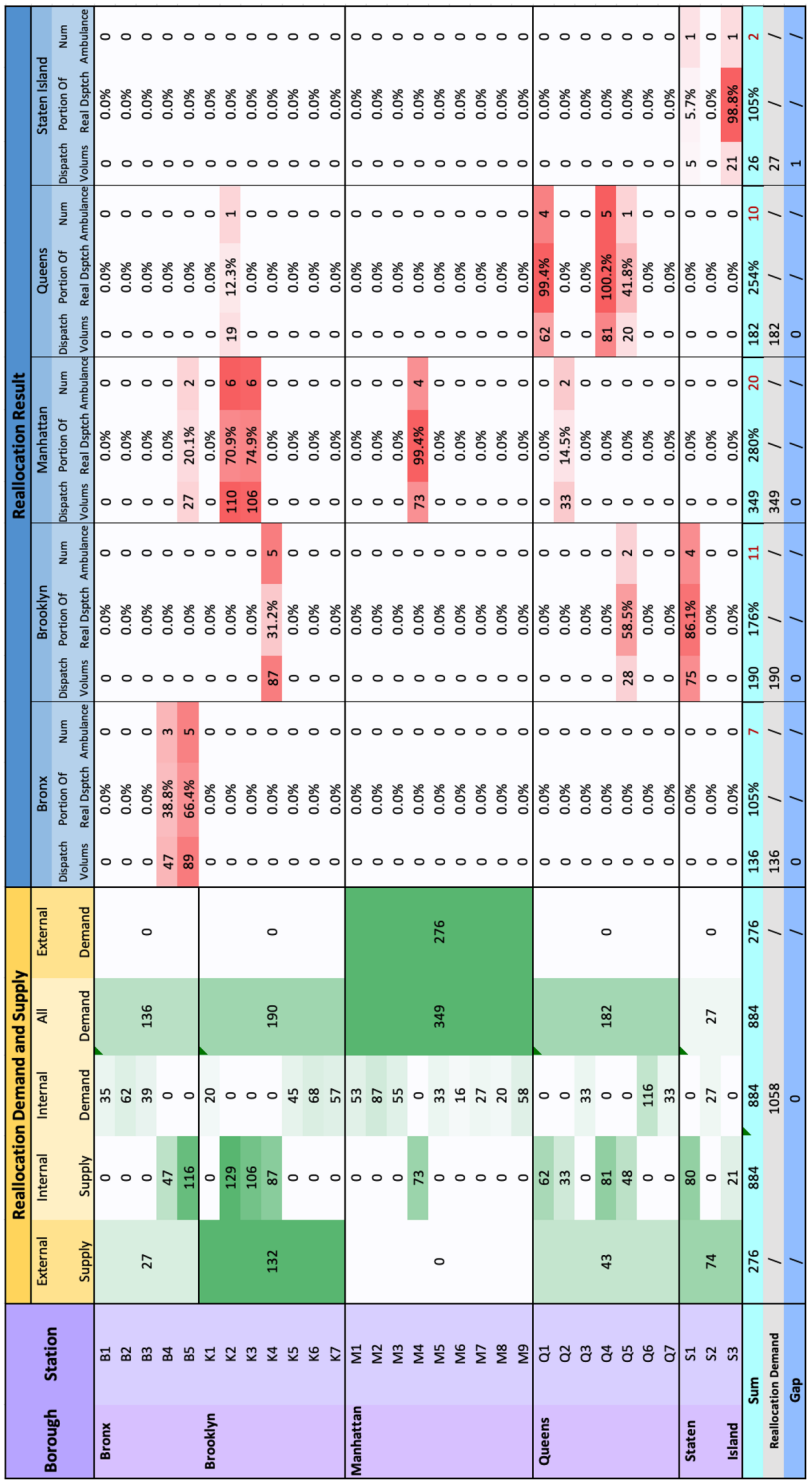
Reallocation demand and supply based on the difference allocation result and real dispatch obtained in Fig. 8. Reallocation result. Reallocation as a percentage of real dispatch, and its portion of estimated capacity

We are able to determine which stations are lower than the dispatch demand that should increase the dispatch capacity and which stations have exceeded the dispatch demand and can supply to move out, based on the dispatch allocation of $EMS\_RR$ model in Sect. 4.3. The average daily difference values between our model and real dispatch are shown in Fig. 8. For the column “2020 Difference”, positive value indicates that the optimized allocation is greater than the real allocation, which implies that the reallocation demand is greater than 0, as well as that the reallocation supply is equal to 0 — “No Supply”, as shown in Fig. 10 “Internal demand”. Negative value indicates that the optimized allocation dispatch is less than the real dispatch, which implies that the reallocation supply is greater than 0, as well as that the reallocation demand is equal to 0 — “No Demand”, as shown in Fig. 10 “Internal supply”. In the same way, a positive sum value of a borough implies that the borough reallocation demand is greater than 0, as well as that the borough reallocation supply is equal to 0 as “External demand”. A negative sum value of a borough implies that the borough reallocation supply is greater than 0, as well as that the borough reallocation demand is equal to 0, as “External supply”. “Borough Demand” indicates the sum of reallocation demand for each borough. We use “Internal supply” and “Borough Demand” as input parameters $p_k$ and $r_i$ in the EMS_RR model.

In Fig. 10, column “Dispatch” indicates the reallocation dispatch from stations to boroughs in $EMS\_RR$ model. “Portion of Real Dispatch” indicates the reallocated dispatch volume per station as a percentage of the real dispatch volume. The column “Num ambulance” indicates the number of ambulances a station should reallocate to a borough, which is inferred by multiplying the “Portion” and the “Estimated Num Ambulances”. (“Estimated Num Ambulances” indicates the “Estimated EMS Num(17.5)” in Fig. 9). The total reallocated dispatch volume is 884, and the total number of reallocated ambulance is 51.

## Conclusion and Future Work

In this work, we present an important problem of long response time for callers caused by inadequate ambulance resources in response to the intense increase in ambulance demand during the initial phase of COVID-19 in New York City. We decompose this into three parts using mathematical planning models and simulation analysis to analyze New York EMS operations. The main purpose is to understand and infer the EMS resource allocation system and reasonably allocate-reallocate EMS resources so that callers (patients) wait times are within acceptable thresholds (7 min in general). We propose a framework jointly considering EMS resource allocation and response time. We demonstrate easy-to-interpret results by using real data collected from public data in New York City, which helps support EMS resource allocation decisions, and our hypothesized results are very close to the real cases. Our study is New York City-specific and observational, but our framework is still general enough to allow for adapting and deriving results for different scenarios. We do not discuss the uncertainty about demand by assuming that all demand is known in advance, thus we do not discuss the predictability of resource demand in the case of a sudden disaster. It may make our conclusions more practical if ambulance demand prediction is also included in the discussion. Also, we do not discuss transfer costs, new ambulance costs, parking costs, especially in cities like New York with expensive parking costs. In addition, we did not analyze backup stations and backup ambulances in our framework. In our analysis, we removed extreme cases and always minimized resources without considering backup plans. Since there are many extreme cases in reality, EMS backup plans could be crucial. When analyzing ambulance demand, priority can be set for demand based on the sensitivity of patient status to response time. If the urgency of the patient can be more accurately identified, EMS will tend to dispatch higher priority callers first in a state of resource scarcity, making it more conducive to resource allocation. However, it is difficult to determine accurately in advance the severity level of a caller’s condition as EMS operators need to understand remotely the patient’s status when calling and the caller needs to dictate the situation, which may lead to inaccurate and incomplete judgment of the severity. Some callers may give up waiting for an ambulance, resulting in a waste of resources as well. Thus, the operator’s judgment at the beginning has a critical role in the overall EMS operation. In the future, it is possible to discuss more specific status of the caller and other uncertainties in our framework. We may also adopt the concepts of our proposed approach and results for application to other agencies that are resource-poor and sensitive to response time.

## Figures and Tables

**Figure:**
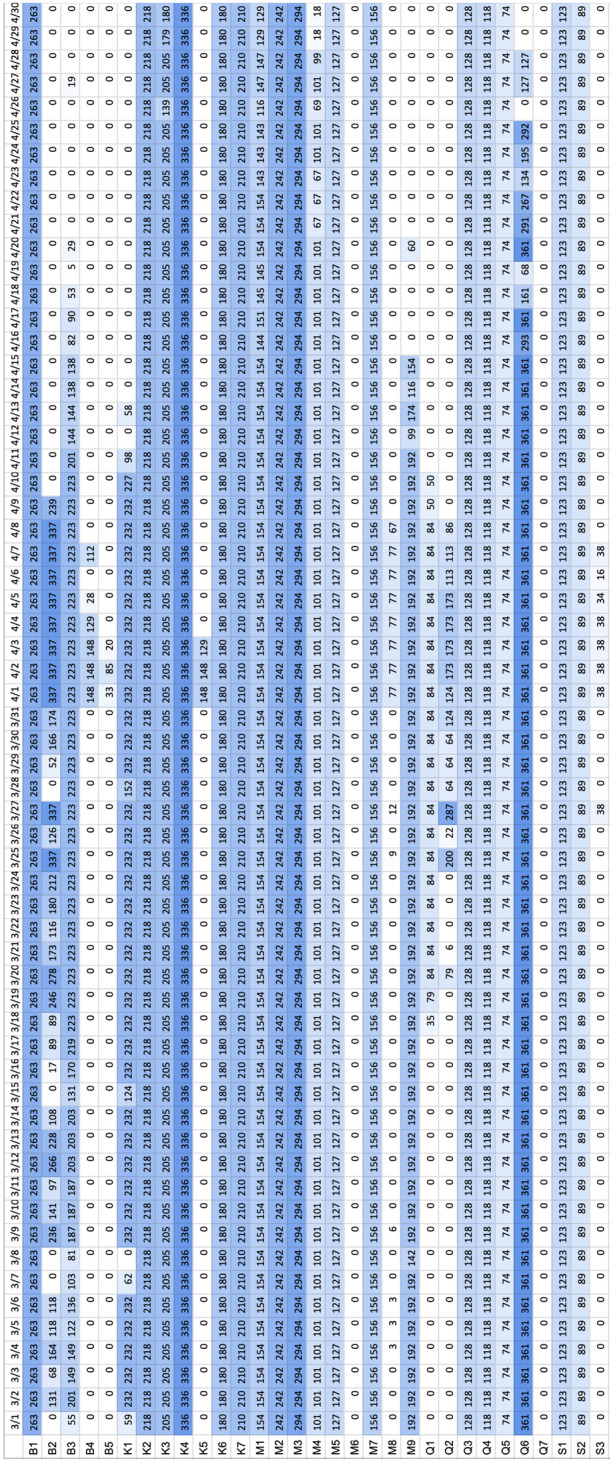
**Fig. A11** Dispatch allocation using travel time 2020

**Figure:**
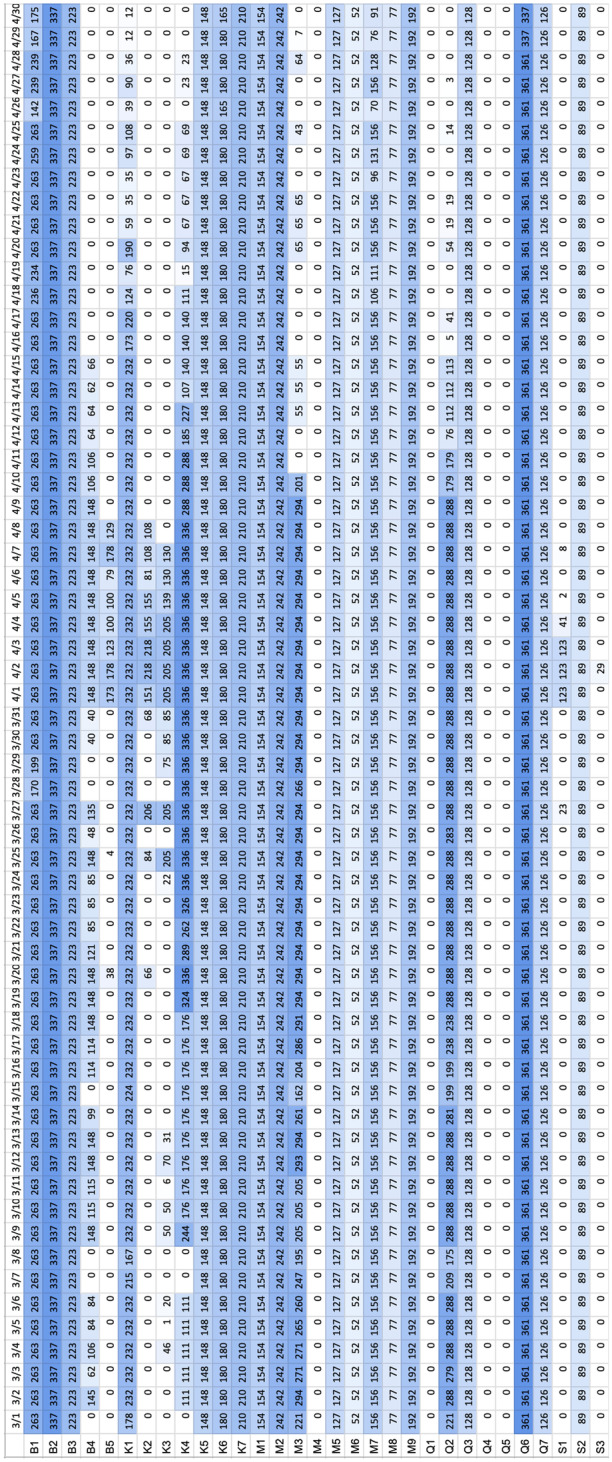
**Fig. A12** Dispatch allocation using driving duration

**Figure:**
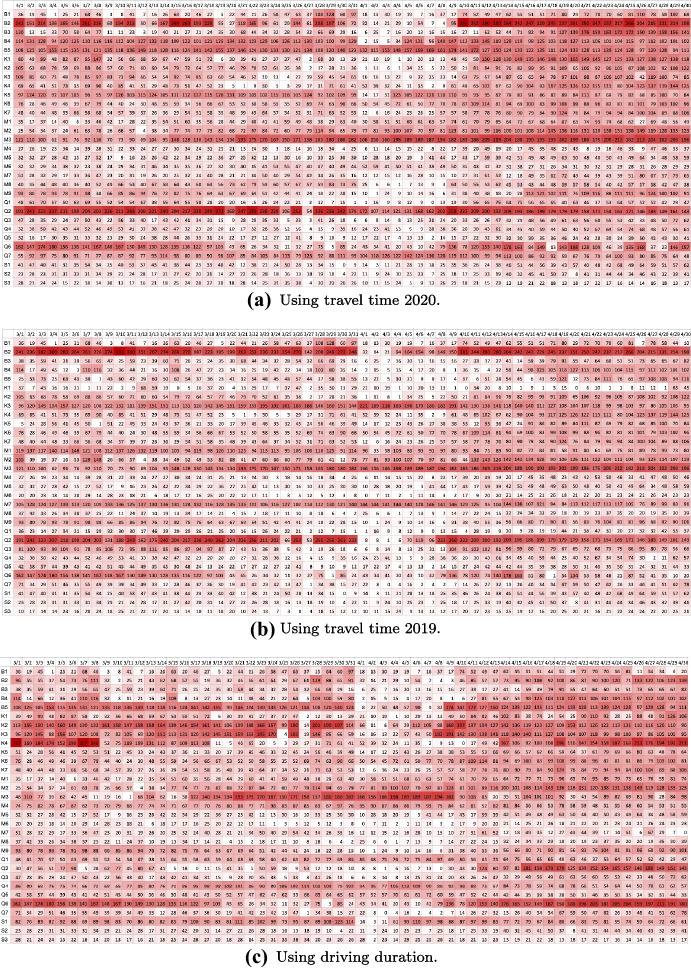
**Fig. A13** Difference dispatch between our model and real dispatch

## Data Availability

The data processed by authors will be shared upon request.

## Appendix

**Table:** **Table A16** Driving distance (mi)

	B1	B2	B3	B4	B5	Q1	Q2	Q3	Q4	Q5	Q6	Q7	S1	S2	S3	
Bronx	**2**.**0**	**4**.**2**	**2**.**7**	**6**.**6**	**2**.**8**	24.0	14.8	13.6	12.4	14.4	10.2	10.6	34.0	33.9	40.2	
Brooklyn	18.5	17.7	19.2	21.2	18.8	13.9	13.6	8.2	8.9	10.9	8.1	15.2	16.5	19.4	25.8	
Manhattan	9.0	8.2	11.7	15.6	9.4	33.0	23.8	22.5	9.3	22.0	5.8	19.6	25.5	25.3	31.7	
Queens	14.7	16.7	11.7	13.8	15.5	**12**.**6**	**6**.**5**	**1**.**7**	**3**.**5**	**3**.**9**	**8**.**9**	**7**.**1**	28.8	31.8	38.2	
Staten Island	32.6	31.8	35.3	39.2	33	26.4	31.6	30.7	25.8	31.4	25	32.2	**3**.**8**	**1**.**0**	**7**.**9**	

	K1	K2	K3	K4	K5	K6	K7	M1	M2	M3	M4	M5	M6	M7	M8	M9
Bronx	28.6	22.8	19.1	17.1	15.8	16.4	13.7	14.2	12.1	11.7	8.3	7.2	7.1	6.0	5.4	4.9
Brooklyn	**6**.**0**	**3**.**8**	**2**.**3**	**4**.**6**	**1**.**6**	**2**.**2**	**3**.**5**	5.0	8.0	8.1	10.7	12.4	13.1	14.1	14.8	18.0
Manhattan	14.7	13.4	13.2	26.1	11.7	8.3	9.6	**5**.**8**	**3**.**1**	**2**.**4**	**2**.**6**	**2**.**4**	**4**.**0**	**11**.**2**	**5**.**2**	**5**.**9**
Queens	17.3	20.9	8.1	6.1	8.0	14.4	11.8	15.3	14.2	13.2	15.7	14.6	16.1	13.1	13.8	16.9
Staten Island	15.2	13.9	19.4	25.0	20.2	16.7	22.1	19.6	24.3	23.5	25.4	27.2	32.6	29.4	32.8	30.4

**Table:** **Table A17** Low priority median travel time in 2020

	B1	B2	B3	B4	B5	Q1	Q2	Q3	Q4	Q5	Q6	Q7	S1	S2	S3	
BRONX	**5**.**57**	**5**.**78**	**6**.**21**	**6**.**18**	**6**.**24**	0	13.13	0	3.43	4.18	6.73	7.97	10.73	0	17.8	
BROOKLYN	8.85	9.38	9.6	0	0	0	28.62	2.03	4.3	12.13	5.73	13.4	4.9	3.27	14.87	
MANHATTAN	2.27	18.28	13.68	0	7.05	0	15.62	18.75	11.6	0	12.4	9.22	3.15	5.18	0	
QUEENS	10.8	7.27	4.45	0	0	**5**.**24**	**5**.**44**	**6**.**13**	**6**.**22**	**6**.**11**	**6**.**22**	**5**.**87**	0	19.75	0	
RICHMOND / STATEN ISLAND	0	20.58	0	0	0	0	0	0	0	0	0	0	**6**.**19**	**7**.**05**	**5**.**07**	

	K1	K2	K3	K4	K5	K6	K7	M1	M2	M3	**M4**	**M5**	M6	M7	M8	M9
BRONX	15.13	11.82	0	7.55	10.9	8.48	0	9.7	2.37	9.5	8.35	0.28	15.3	3.57	12.7	6.28
BROOKLYN	**6**.**42**	**5**.**13**	**5**.**73**	**6**.**37**	**7**.**32**	**4**.**88**	**6**.**42**	7.9	3.28	8.97	6.73	7.82	0	9.9	10.05	0
MANHATTAN	10.12	7.67	5.47	5.57	12.2	3.77	8.1	**5**.**81**	**5**.**53**	**5**.**15**	**6**.**31**	**5**.**88**	**8**.**15**	**6**.**73**	**6**.**89**	**5**.**88**
QUEENS	7.13	4.55	8.93	2.15	6.77	10.63	3.65	0	4.12	5.28	3.8	11.85	0	6.55	0	9.93
RICHMOND / STATEN ISLAND	11.48	3.35	19.47	0	0	8.47	28.52	1.62	0	4.7	0	7.41	0	0	0	0

**Table:** **Table A18** High priority median travel time in 2020

	B1	B2	B3	B4	B5	Q1	Q2	Q3	Q4	Q5	Q6	Q7	S1	S2	S3	
BRONX	**5**	**2**.**74**	**7**.**05**	**5**.**87**	**2**.**47**	0	0	0	0	0	0	0	0	0	0	
BROOKLYN	0	0	0	0	0	0	0	0	4.97	0	0	0	3.35	0	0	
MANHATTAN	0	0	0	0	0	0	0	0	0	3.97	0	0	0	0	0	
QUEENS	0	0	0	0	0	**5**.**32**	**3**.**77**	**8**.**38**	**5**.**03**	**2**.**98**	**4**.**5**	**3**.**67**	0	0	0	
RICHMOND / STATEN ISLAND	0	0	0	0	0	0	0	0	0	0	0	0	**6**.**18**	**5**.**08**	**0**.**6**	

	K1	K2	K3	K4	K5	K6	K7	M1	M2	M3	**M4**	**M5**	M6	M7	M8	M9
BRONX	0	0	4.62	0	0	0	0	0	0	3.85	0	0	0	0	0	0
BROOKLYN	**2**.**78**	**3**.**58**	**2**.**6**	**3**.**84**	**2**.**3**	**3**.**1**	**4**.**46**	0	0	0	0	0	0	0	0	0
MANHATTAN	0	0	0	0	0	1.37	0	**1**.**8**	**2**.**5**	**2**.**31**	**2**.**99**	**3**.**67**	**0**.**65**	**3**.**01**	**2**.**2**	**1**.**31**
QUEENS	0	0	0	0	0	0	0	0	0	0	0	0	0	0	0	0
RICHMOND / STATEN ISLAND	0	0	0	0	0	6.6	0	0	0	0	0	0	0	0	0	0

**Table:** **Table A19** Median travel time in 2019

	B1	B2	B3	B4	B5	Q1	Q2	Q3	Q4	Q5	Q6	Q7	S1	S2	S3	
BRONX	**7**.**66**	**8**.**43**	**8**.**46**	**8**.**07**	**8**.**00**	8.22	0.00	0.00	0.00	12.32	15.22	17.02	0.00	0.00	7.52	
BROOKLYN	15.33	14.22	16.33	0.00	7.55	4.15	7.27	18.87	6.30	0.00	7.02	4.95	5.52	10.22	0.00	
MANHATTAN	6.07	6.48	2.05	6.47	15.92	11.42	9.83	6.52	0.00	0.00	5.18	13.67	7.48	4.38	0.00	
QUEENS	0.00	10.45	6.97	0.00	0.00	**5**.**45**	**7**.**68**	**7**.**03**	**7**.**42**	**6**.**32**	**6**.**80**	**6**.**82**	4.10	0.00	0.00	
RICHMOND / STATEN ISLAND	0.00	9.78	0.00	0.00	0.00	0.00	0.00	0.00	3.88	0.00	0.00	0.00	**6**.**45**	**6**.**30**	**7**.**42**	

	K1	K2	K3	K4	K5	K6	K7	M1	M2	M3	**M4**	**M5**	M6	M7	M8	M9
BRONX	11.30	19.58	27.30	8.02	10.58	8.60	7.22	21.85	17.72	5.35	13.70	22.67	0.00	13.12	0.00	9.03
BROOKLYN	**7**.**27**	**5**.**78**	**8**.**58**	**8**.**92**	**7**.**03**	**7**.**35**	**7**.**78**	15.52	7.22	10.27	2.22	2.98	2.42	0.00	2.12	5.23
MANHATTAN	24.38	1.82	13.67	17.62	10.37	9.23	8.68	**6**.**65**	**6**.**27**	**6**.**85**	**7**.**58**	**6**.**95**	**8**.**09**	**8**.**95**	**10**.**87**	**7**.**63**
QUEENS	11.68	8.15	11.45	2.17	0.00	3.42	5.08	17.80	21.17	8.03	7.03	10.23	0.00	0.00	0.00	25.32
RICHMOND / STATEN ISLAND	2.92	14.53	0.00	12.47	0.00	2.00	11.33	16.55	0.00	0.00	0.00	0.00	0.00	0.00	0.00	0.00

**Table:** **Table A20** Cancel rate

Area	Daily	Area	Daily
B1	94.39%	K1	92.67%
B2	95.17%	K2	91.02%
B3	96.15%	K3	94.97%
B4	95.60%	K4	95.33%
B5	95.17%	K5	94.42%
Q1	91.63%	K6	90.99%
Q2	95.19%	K7	93.49%
Q3	95.36%	M1	91.79%
Q4	94.73%	M2	90.58%
Q5	94.30%	M3	89.59%
Q6	94.79%	M4	94.45%
Q7	94.65%	M5	93.66%
S1	90.44%	M6	94.15%
S2	93.30%	M7	94.73%
S3	92.17%	M8	95.38%
		M9	94.96%

**Table:** **Table A21** Parameter of normal distribution for service time in March and April 2020

Area	Mean	Standard Deviation	Area	Mean	Standard Deviation
B1	55.47	11.12	K1	52.24	11.87
B2	54.24	9.39	K2	50.29	12.50
B3	58.37	11.24	K3	57.04	14.35
B4	57.31	13.84	K4	58.38	10.57
B5	56.27	13.35	K5	55.01	16.36
Q1	50.13	19.23	K6	51.75	14.72
Q2	55.27	10.58	K7	54.83	14.04
Q3	52.91	16.66	M1	50.06	15.61
Q4	55.36	16.91	M2	47.08	12.26
Q5	53.47	19.60	M3	47.62	13.58
Q6	53.81	10.71	M4	54.55	16.84
Q7	53.74	16.10	M5	52.68	15.23
S1	47.08	15.79	M6	55.60	20.43
S2	52.56	18.30	M7	54.11	13.80
S3	50.94	22.59	M8	54.70	19.22
			M9	55.67	13.34

## References

[1] Aringhieri R, Bruni ME, Khodaparasti S, van Essen JT (2017). Emergency medical services and beyond: addressing new challenges through a wide literature review. Comput Oper Res.

[2] Hsia RY, Huang D, Mann NC, Colwell C, Mercer MP, Dai M, Niedzwiecki MJ (2018). A US National Study of the Association between income and ambulance response time in cardiac arrest. JAMA Netw Open.

[3] Alvarado S (2020) What is the average emergency response time? https://medicalnewsbulletin.com/response-time-emergency-medical-services/

[4] Prezant DJ, Lancet EA, Zeig-Owens R, Lai PH, Appel D, Webber MP, Braun J, Hall CB, Asaeda G, Kaufman B (2020). System impacts of the COVID-19 pandemic on New York City’s emergency medical services. J Am Coll Emerg Physicians Open.

[5] Edelman S (2020) New Yorkers are dying as ambulance response times surge amid Coronavirus

[6] Kaufman M (2020) Ambulance response times surge in Queens amid pandemic: report

[7] Bürger A, Wnent J, Bohn A, Jantzen T, Brenner S, Lefering R, Seewald S, Gräsner J-T, Fischer M (2018). The effect of ambulance response time on survival following out-of-hospital cardiac arrest: an analysis from the German resuscitation registry. Dtsch Arztebl Int.

[8] Alumran A, Albinali H, Saadah A, Althumairi A (2020). The effects of ambulance response time on survival following out-of-hospital cardiac arrest. Open Access Emerg Med: OAEM.

[9] Improving Ambulance Response Time (2007) High impact changes and response times algorithms for NHS ambulance trusts. Department of Health

[10] Farahani RZ, Lotfi MM, Baghaian A, Ruiz R, Rezapour S (2020) Mass casualty management in disaster scene: a systematic review of or & MS research in humanitarian operations. Eur J Oper Res

[11] Marla L, Krishnan K, Deo S (2021). Managing EMS systems with user abandonment in emerging economies. IISE Transactions.

[12] Simpson NC, Hancock PG (2009). Fifty years of operational research and emergency response. J Oper Res Soc.

[13] Singer M, Donoso P (2008). Assessing an ambulance service with queuing theory. Comput Oper Res.

[14] Acuna JA, Zayas-Castro JL, Charkhgard H (2020). Ambulance allocation optimization model for the overcrowding problem in US Emergency Departments: a case study in Florida. Socio Econ Plan Sci.

[15] Ahmadi G, Tavakkoli-Moghaddam R, Baboli A, Najafi M (2020) A decision support model for robust allocation and routing of search and rescue resources after earthquake: a case study. Oper Res 1–43

[16] Berkoune D, Renaud J, Rekik M, Ruiz A (2012). Transportation in disaster response operations. Socioecon Plann Sci.

[17] Khalili-Damghani K, Tavana M, Ghasemi P (2021) A stochastic bi-objective simulation–optimization model for cascade disaster location-allocation-distribution problems. Ann Oper Res 1–39

[18] Boutilier JJ, Chan TCY (2020). Ambulance emergency response optimization in developing countries. Oper Res.

[19] Bélanger V, Lanzarone E, Nicoletta V, Ruiz A, Soriano P (2020). A recursive simulation-optimization framework for the ambulance location and dispatching problem. Eur J Oper Res.

[20] Wang J, Wang Y, Yu M (2020) A multi-period ambulance location and allocation problem in the disaster. J Comb Optim 1–24

[21] Cookson R, McCabe C, Tsuchiya A (2008). Public healthcare resource allocation and the rule of rescue. J Med Ethics.

[22] Doan XV, Shaw D (2019). Resource allocation when planning for simultaneous disasters. Eur J Oper Res.

[23] Jagtenberg CJ, van den Berg PL, van der Mei RD (2017). Benchmarking online dispatch algorithms for emergency medical services. Eur J Oper Res.

[24] Fire Department of New York City (FDNY) (2021) EMS incident dispatch data: NYC open data

[25] Alexander M (2018) Reviving EMS: restructuring emergency medical services in New York City. https://cbcny.org/research/reviving-ems

[26] Fire department of the city of New York. https://fire.fandom.com/wiki/Fire_Department_of_the_City_of_New_York

